# Menstrual Health Problems of Women Indigenous Peoples around Protected Forest Area in Sumatra, Indonesia, and Plants’ Usefulness to Treat It

**DOI:** 10.34763/jmotherandchild.20222601.d-22-00064

**Published:** 2023-06-11

**Authors:** Yesi Mustika Sari, N. Novriyanti

**Affiliations:** Universitas Adiwangsa Jambi. Jl. Sersan Muslim No.RT 24, The Hok, Kec. Jambi Sel., Kota Jambi, Jambi, Indonesia; Forestry Department, Faculty of Agriculture, Universitas Lampung. Jl. Prof. Dr. Soemantri Brojonegoro No.1 Gedong Meneng, Rajabasa, Lampung, Indonesia 361361; KKI Warsi, Jl. Inu Kertapati No. 12 Pematang Sulur, Telanaipura, Kota Jambi, Indonesia

**Keywords:** adolescents, menstruation, Orang Rimba, ethnobotany, gender health

## Abstract

**Background:**

Some cultures have a favorable view of menstruation, consider it sacred, and respect the female body, so some local wisdom and the practice of using plant species are also attached to it. Moreover, menstruation is an integral part of reproductive health for women as mothers of a nation. However, the management of menstrual problems included in the United Nations Sustainable Development Goals (gender justice) goals in several indigenous communities around the forest has not received attention.

**Objective:**

This study aims to explain the situation of menstrual management, predict indications of reproductive problems, and record the practice of using plants to overcome these problems in indigenous tribal communities around the forest.

**Material and methods:**

A total of 15 youths of the Orang Rimba, one of the marginal indigenous people in Jambi Province, Sumatra Island, Indonesia, were the subjects of measurement of all variables using anthropometric measurement procedures. The 15 girls were also interviewed regarding menstrual problems, personal hygiene management, and using plant species to overcome them. Meanwhile, ten adults became respondents to the complementary primary data.

**Results:**

No plant species were explicitly used to treat menstrual problems. Four species are used by the Orang Rimba concerning labor management (pre- and postpartum).

**Conclusion:**

There are no significant reproductive problems despite the incidence of dysmenorrhea. However, aspects of nutrition and personal hygiene, including during menstruation, still need special attention, especially considering that the typology of Orang Rimba varies according to their Tumenggung and the characteristics of their forest habitat; It is challenging to measure their health as a group. This condition may also apply to other communities around the forest due to their limited reproductive health knowledge.

## Introduction

The use of various plant species to maintain the health of individuals and groups of people has been widespread ever since before the Christian era. A review of the history of using plants as health maintainers and healers of disease found records of this activity dating back to the ancient Egyptians in the Himalayan lands [1]. All forms of pain the body responds to can be treated with potent substances extracted from plants [2], including using plants to overcome various reproductive health problems.

Reproductive health is important for women, especially adolescents, both in cities and for those living in villages. The status of women's reproductive health since adolescence will affect their health conditions in the future [3]. One of the indicators of achieving reproductive health is healthy and regular menstruation (periodic and cyclic bleeding originating from the uterus that is physiological in nature and accompanied by the shedding of the endometrial wall) [4]. Awareness of good menstrual health is necessary [5]. Achieving gender equity in health, such as reproductive health, is also included in one of the United Nations Sustainable Development Goals (SDGs 3.7, 5.6, and 6.2).

Unfortunately, many menstrual events are usually accompanied by headaches, specifically migraines that require more doses of medication or treatment efforts, especially when accompanied by nausea [6]. Severe menstruation can be one of the triggers of death because those who experience menstruation do not realize that there is iron deficiency anemia that follows it. Menstruation management in developing countries is reported to be poor [7] because of the problem of poor hygiene in the place of residence, and lack of knowledge and handling during menstruation [8,9,10]—not to mention whether abdominal pain during menstruation is accompanied by emotional problems that occur in women [11,12]. Adolescents’ ability to understand cycles is still a problem in rural and urban areas [13]. How do teenagers with the typology of indigenous people living around the forest with limited access to health facilities deal with the problems of menstrual hygiene and health? What is the role of local knowledge in the practice of using plant species to solve this problem?

The use of plants to overcome various reproductive health problems in indigenous communities near forests has been reported by many countries in the world. In Nigeria, 56 species of plants treat menstrual issues, which are usually also associated with postpartum problems [14]. Indigenous people in India use 71 plant species to solve the problem of irregular menstruation [15]. In Indonesia, herbal medicine has indeed been known as an effort to maintain health, including when there are reproductive problems in women, especially for traditional and some urban communities on the island of Java [16,17] or some areas with Javanese ethnic communities [18]. Until now, few scientific reports on using plant species to treat women's reproductive health problems, especially regarding menstruation in forest edge community groups in Sumatra, Indonesia. Many community groups are still far from having complete information, even though they are not far from a health service centre. So, this study tries to explain the situation of menstrual management, suspect indications of reproductive problems, and record the traditional practice of using plants to overcome these problems in indigenous communities around the forest.

## Material and Methods

### Study Site and Sample

The sample test used in this study was the female population of the Orang Rimba, who live in the Bukit Duabelas National Park (TNBD) area. Geographically, TNBD is located at 1°51′0″S and 102°52′0″E, administratively in Sarolangun Regency, Jambi Province, Indonesia. In this protected area, the Orang Rimba live in small groups led by a Tumenggung. Tumenggung is accompanied by a Menti (customary court judge) and ninik Mamak (customary leaders) in making decisions regarding the Orang Rimba [19]. After going through negotiations and passing their Tumenggung and ninik Mamak permits, the number of samples was determined first. As many as 25 women of the Orang Rimba were willing to measure with free, informed consent (FPIC) of this prospective human sample. In addition, all test variables on the human selection in this study also passed the ethics test.

The 25 females of the Orang Rimba came from two groups: 12 people from the Tumenggung Ngrip group who lived within the working area of Air Hitam 1 TNBD Resort, and the rest from the Terab Group at Batin 24 or 1A Hajran Resort of TNBD (Table 1). These groups represent the following conditions:

- located close to state-run areas (i.e., national parks) and privately managed areas (rubber plantations);
- far or close to community settlements; and
- can access community health service centres.

### Data and Sampling Measurement

Nutritional status was obtained through an anthropometric measurement approach, which was then categorized based on body mass index (BMI). Anthropometric measurements (height and weight) were carried out according to World Health Organization (WHO) standards. Height was measured using a stadiometer and recorded to the nearest 0.1 cm. During the measurement, the protruding body parts of the girl (occipital, shoulder, buttocks, and heels) touched the stadiometer; the shoes were removed, and she stood in Frankfurt's position. Weight was measured with a glass weight scale (GEA brand) and recorded to the nearest 0.1 kg. Heavy clothing and shoes were removed [20].

**Table 1.** Sampling number of females of the Orang Rimba who have gone through previous FPIC.

**No.**	**Group of the Orang Rimba**	**Adolescent**	**Adult**	**Total**
1	Tumenggung Ngrip at Air Hitam 1 Resort of TNBD	5	7	12
2	Terab at Batin 24/1A Hajran Resort of TNBD	10	3	13
				25

Indications of adolescent reproductive problems are known through a personal approach. The subjects studied have done a private interview about the incidence of menstruation and personal hygiene management. These two data types are broken down as follows:

- Menstrual events include menstrual cycle patterns, length of menstrual days, menarche (first menstruation), and abnormalities that cause discomfort during menstruation (such as dysmenorrhea).
- Personal hygiene management includes the frequency of self-cleaning and the use of cleaning tools.

After the physical measurements were completed, the subjects studied were gathered in small groups to be interviewed openly but exclusively. ‘Exclusive’ means that only women from the Orang Rimba attend the interview activities. This activity aims to explore the practice of using plant species to improve reproductive health as measured by the level of community knowledge on the use of various plant species to maintain women's reproductive health, not limited to menstrual problems. The same interview technique as various previous studies in exploring social phenomena related to community interactions around the forest [18,19,21] was also used in this study.

### Data analysis

All data collected were tabulated in Microsoft Excel. The list of plant species was grouped according to their designation, which is presented in the table. The collected anthropometric data will be analysed for BMI value through the formulation (F1) according to the WHO-Asia BMI Classification [22]. Furthermore, the data are illustrated through a diagram.

(F1) $$\mathrm{BMI}=\frac{\mathrm{weight}\left(\mathrm{kg}\right)}{\mathrm{height}{\left(M\right)}^{2}}$$

Description:

BMI <18.5: underweight
BMI 18.5 up to 22.9: normal
BMI >22.9: overweight

## Results and Discussion

### Research Subject Situation and Application of Orang Rimba Customs in Research Activities

**Figure 1.**
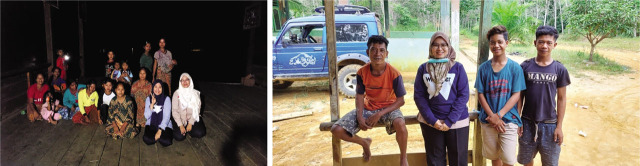
[left] Documentation with the female Orang Rimba in the Tumenggung Ngrip Group, while in the Terab Group, it can only be done with the male Orang Rimba [right].

Each member of the Orang Rimba group in Jambi Province, Indonesia, has the same characteristics and customs. Still, for those already associated with other communities in the village, some regulations can adapt to the situation, and vice versa for the Orang Rimba “in” to remain steadfast in any case. This affects the amount of data and documentation obtained. Taking photos and videos at the Tumenggung Ngrip Group was possible because Tumenggung and the Orang Rimba traditional leaders had given permission (see Figure 1). On the other hand, the Tumenggung of the Orang Rimba Terab Group did not permit the females of the Orang Rimba to be highlighted by the camera; fortunately, during the interview with the Tumenggung and the male Orang Rimba, the interview was allowed to be documented.

**Figure 2.**
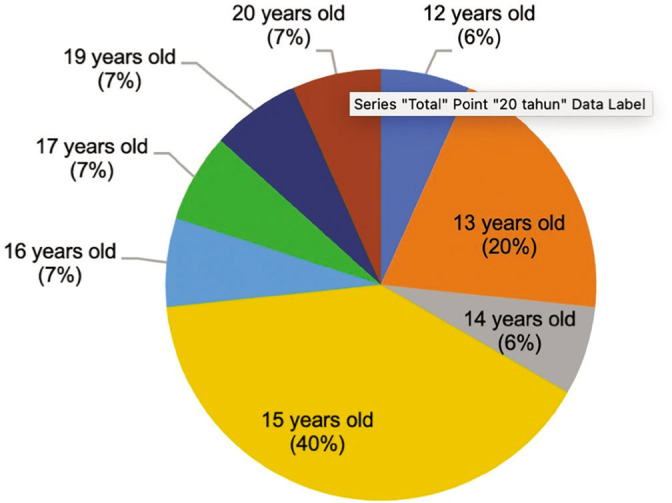
Estimated age of Orang Rimba adolescents in Bukit Dua Belas National Park, Jambi Province, as research subjects.

The age of the respondents in this study is difficult to measure with certainty. The subjects studied only mentioned age through estimates. No one can clearly recognize the year of birth because they do not know the calendar system. Based on this situation, as many as 46% of the young Orang Rimba respondents in this study were in the middle adolescent group. Of the 15 adolescents studied, the age distribution ranges from 12 to 20 years (see Figure 2).

### Nutritional Status of Research Subjects

**Figure 3.**
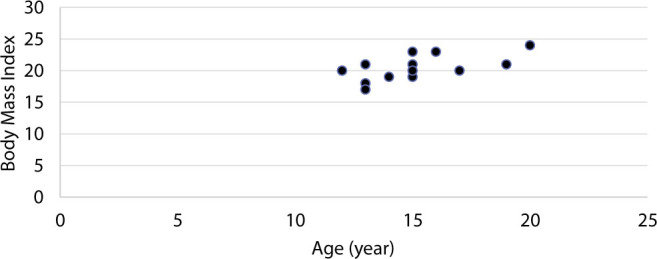
Distribution of body mass index by the Age of Orang Rimba Women.

Adolescence is a critical development period, and nutritional status during adolescence affects health in adulthood and old age. However, adolescent health has not received widespread attention, especially among adolescent girls, including the Orang Rimba. As many as 38% of the Orang Rimba adolescent girls younger than 17 (n=13) experience nutritional imbalances. In adolescent girls with the most frequent age, namely, 15 years, two people are overweight (see BMI distribution in Figure 3). However, as many as 60% of those studied fall into the normal BMI category. The findings in this study relate to previous nutrition reports, especially in the Terab Group, where the number of toddlers experiencing malnutrition is still below 15% [23].

However, the strenuous physical activities undertaken by young Orang Rimba girls, such as housework (cooking, washing, and looking for food), lifting water from rivers, and looking for firewood, can cause fatigue and interfere with their health. Adolescents who experience disharmony BMI are vulnerable to this. Adolescent girls with a normal but quite low BMIs are also at risk if their daily physical activity and eating patterns are not maintained.

### Menstrual Occurrence

Menstruation in the everyday language of the Orang Rimba is known as “kelu kuto.” None of the female Orang Rimba pays special attention to how menstruation occurs, whether it is the pattern, the length of the day, or the menarche. Orang Rimba women can still recognize the length of the menstrual period because, in Orang Rimba terms, this is called “malom men,” calculated from the first day of menstrual blood coming out until it is finished. The average length of menstruation in juvenile Orang Rimba is five to six days, with a maximum number of days reaching eight. Normally, women experience bleeding within three to five days each month [24]. However, given that the Orang Rimba does not know the calendar system, the pattern of the menstrual cycle and menarche (first menstruation) is also difficult to define with certainty. The menstrual cycle pattern is one of many important physiological rhythms, the distance between the start of the last menstruation and the next menstruation with a normal cycle of 25–32 days [24]. Menstrual cycle length is an important indicator of gynaecological disorders such as endometriosis and PCOS, as well as reproductive parameters, including age at menopause and fertility [25].

Ignorance of the pattern or menstrual cycle can be difficult because it is difficult to identify the regularity of the process, which is sometimes accompanied by dysmenorrhea. Dysmenorrhea is pain that occurs with menstruation without obvious pelvic involvement at the start of menstruation and occurs more often before the age of 20 years [26]. From the data obtained, most female Orang Rimba adolescents in this study (73%) experienced dysmenorrhea from low back pain, stomach pain, and headaches during menstruation. 73% are generally in the age range of 14–20 years, and three are teenagers with disharmony BMI (see Figure 3).

As for menarche, it usually occurs between the ages of 10 and 16, with an average age of onset being 12 years [26]. Among Orang Rimba girls, menarche occurs between 11 and 18 years of age, with a mean of 13 years (50%). Overall, menarche in the younger generation of Orang Rimba women occurs later than in adolescents. Research findings also support that as many as 12% of respondents experienced late menarche (age > 15 years). The age at which a woman experiences menarche varies, with genetic and environmental factors such as socioeconomic status, family life, race, exercise, and diet. Menarche tends to be painless and occurs without warning. Menarche signals the beginning of reproductive capacity and is closely associated with the ongoing development of secondary sexual characteristics. However, the onset of menarche does not guarantee ovulation or fertility, although the two are often associated [27]. Of particular concern is menstruation too early (before or at age 10), late (after age 15), or not at all [28]. This issue will have a negative impact in the future.

### Personal Hygiene Management

Adolescents of the Orang Rimba are now familiar with sanitary napkins, which are referred to as “pempos” in the Orang Rimba language. Some of them use it during menstruation. Although sanitary napkins are well known, the use of cloth during menstruation is still mainstream because they do not need to spend money to buy sanitary napkins. This condition is common in developing countries. In rural areas, women do not have access to sanitation products, know little about the types and methods of using them, or cannot afford to buy them because of their high costs [29]. The cleanliness and hygiene of using sanitary cloth napkins is still a question mark, just like the reusable pads currently circulating. Its use is still a common choice because the high leakage factor can cause physical activity inhibition [30]. The Orang Rimba take water directly from the river to meet their water needs. Some of them have settlements near villages; their water source comes from wells. The Orang Rimba bathe one to two times a day. The use of soap is common, so they already know that maintaining cleanliness is good. Unfortunately, there is no effort to clean yourself, specifically when menstruation arrives.

**Figure 4.**
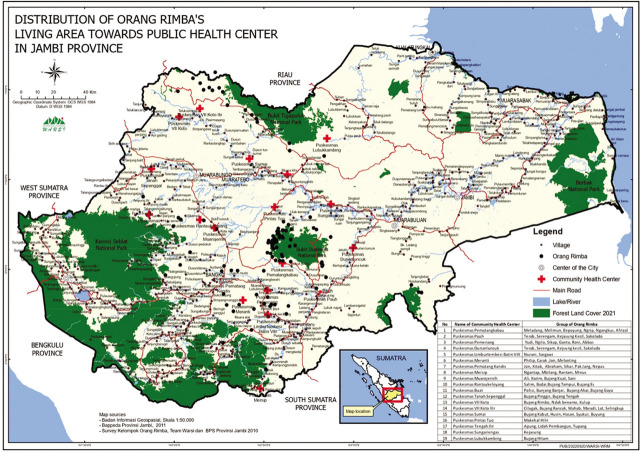
Map of the Distribution of the Orang Rimba in Jambi Province, Sumatra, and Their Accessibility to the Community Health Service Centre (Produced by KKI Warsi).

The Orang Rimba are spread out and are said to be able to access the nearest public health service (PUSKESMAS) (see Figure 4). But the access is not just visiting; the Orang Rimba have only recently been able to get these services and be considered a nation. The marginalization in the Orang Rimba puts them in a state of health that is far from being noticed. Thus, it is natural that it is still difficult to find women of the Orang Rimba who are very clean. Most of the diseases in this community are related to the skin [31]. From this, it is clear that hygiene is a major problem for the Orang Rimba, especially women.

**Table 2.** The uses of plant species related to women's reproductive health.

Species description	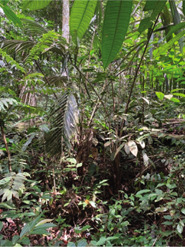	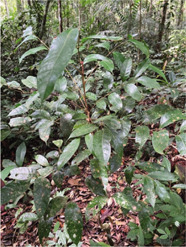	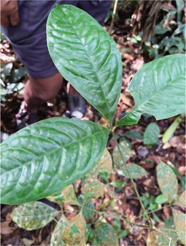	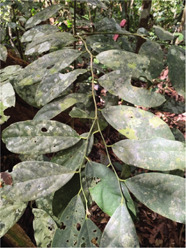
Local name	Ibul	Kedunduk tunjuk	Selusuh	Sentubung
Scientific name	*Korthalsia echinometra*	*Santiria laevigata*	*Luvunga sarmentosa*	*Xanthophyllum vitellinum*
Use	Treating female genital pain after childbirth; can be a cure for infertility	An antidote when postpartum nausea appears; can also treat dysentery	Expedite the release of the baby during childbirth	Facilitate the release of the baby during childbirth
How to use	Stems are boiled, then drunk or eaten directly	The root is cut or the bark is boiled, and drunk	The leaves are boiled and drunk by the mother who is about to give birth	The leaves are boiled and drunk; the leaves are kneaded and applied to the stomach

### Local Wisdom and Useful Plant Species for Women's Reproductive Health

Specifically, there are no certain plants that the Orang Rimba use to treat menstrual problems, such as pain relievers or menstrual-stimulating ingredients in the form of ‘herbal medicine’, as it is commonly known by the Indonesian people [16,17]. Previous research on the Orang Rimba scattered in Tebo Regency also showed similar results; no plant species were used to treat menstrual problems [32,33]. The Orang Rimba, another group in the TNBD area, has also been studied before, none of which reported using certain plant species to treat menstrual problems [31].

Local knowledge of the Orang Rimba regarding useful plant species whose practices relate to aspects of femininity is only available to facilitate labour or drugs to treat postpartum pain. Only four species were identified (Table 2). One of the four has been recognized by the Orang Rimba, who live outside the TNBD area with the same use, namely selusuh (*Luvunga sarmentosa*) [34]. In comparison, the other three species have only been discovered in this study.

**Figure 5.**
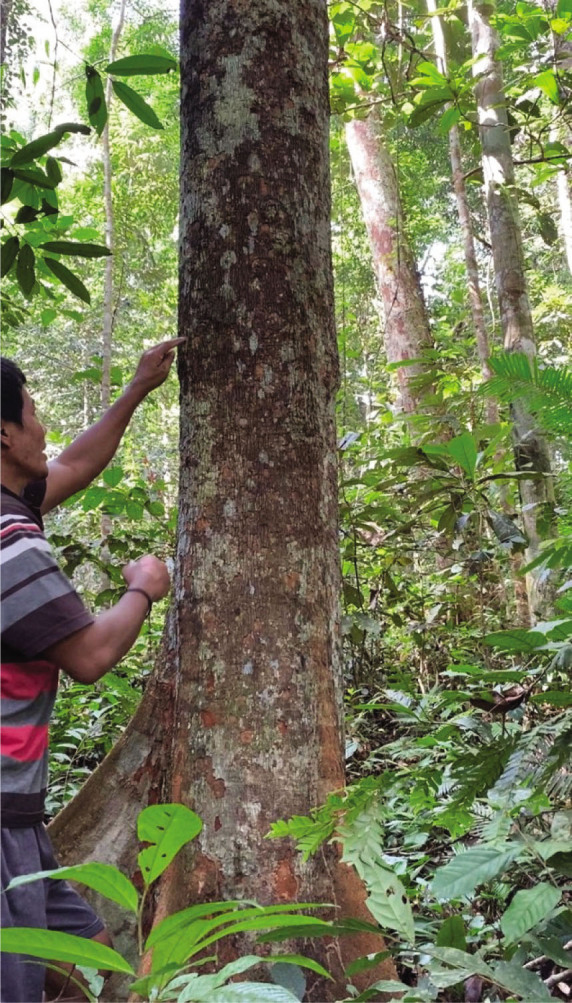
One of the Orang Rimba, who acts as a guardian of wisdom and is an identifier of medicinal plants, is demonstrating the process of marking the birth of a baby on Senggeris wood (*Koompassia excelsa*).

Although no specific plant species were found to treat menstrual problems, the Orang Rimba have wisdom in maintaining the hygiene of menstrual events. During menstruation, terab bark is used as a base to absorb menstrual blood, which is supported using cloth; after use, it is immediately discarded, and the fabric is washed [35]. The Orang Rimba use terab bark (*Artocarpus* sp.) as a *cawat* (pubic cover, resembling underwear, and usually worn by men). The *cawat* is made not only of terab wood but also of the ipuh (*Antiaris toxicaria*) plant. The terab bark was used before they knew sanitary napkins; now, they are no longer used because they are difficult to find. Not only is terab wood hard to find, but the sentubung tree (*Xanthophyllum vitellinum*) presented in Table 2 is also on the verge of extinction. In the Orang Rimba custom, the *sentubung budak* (sentubung tree sapling) will be marked every time a baby is born: one tree only for one baby. Apart from sentubung, other Orang Rimba also believe in Sengeris (*Koompassia excelsa*) (Figure 5) as a birth marker.

Adat is defined as a concept with a high-value system that includes collective beliefs and practices that become the cultural identity of a particular community [36]. In terms of using plants, it is known that the identity of the Orang Rimba is starting to fade. Two important plant species closely related to their customs, namely, birth markers, mean the Orang Rimba must give up some of their traditions because they are not being used. Apart from the fact that there are not many more women in the jungle, many women from the jungle have given birth outside (not in *Susudungon*, where they live in the TNBD area). Those who give birth outside of this are not obliged to mark the birth of a child with that tree species.

Weak customs and damaged forest conditions make the Orang Rimba groups accustomed to living insecurely with change—for example, the Terab group studied. Menstruation is still a sensitive topic to discuss with the Orang Rimba. Some cultures positively view menstruation as sacred and inseparable from cultural rhythms and respect for the female body, while other cultures treat menstruation with ambivalence [37]. Awareness of good menstrual health is needed [5]. Complete and accurate information must be obtained by young women even from an early age so that false beliefs and taboos can be avoided and their self-confidence increases [38,39] so that advocacy for community-based initiatives that focus on improving the welfare of young women in the world through management of healthy and hygienic menstruation [40].

## Conclusion

Although no useful plant species have been found to prevent and overcome female health issues, especially menstrual problems, in general, there is no indication of serious reproductive health problems in one of the indigenous people around the forest, namely the Orang Rimba; it is just that the nutritional aspect needs to be considered. This is important because it can relate to reproductive physiology affecting the future of the Orang Rimba.
